# A New Era for Analytical Cellular Pathology

**DOI:** 10.1155/2020/8502160

**Published:** 2020-01-14

**Authors:** Dimitrios Karamichos

**Affiliations:** ^1^Department of Ophthalmology, Dean McGee Eye Institute, University of Oklahoma Health Sciences Center, Oklahoma City, OK 73104, USA; ^2^Department of Cell Biology, University of Oklahoma Health Sciences Center, Oklahoma City, OK 73104, USA

Dear Readers,

I am delighted and honored to begin my role as Chief Editor for *Analytical Cellular Pathology* (ACP). Please allow me to introduce myself before I outline my vision for our journal. I earned my PhD in Tissue Engineering/Molecular Biology in 2006 at University College London, UK. After completing my PhD, I served as a Postdoctoral Fellow at the University of Texas Southwestern Medical Center and later at the Schepens Eye Research Institute at Harvard Medical School. My research focus was on the development of new therapies to prevent the devastating effects of corneal fibrosis. In 2013, I moved to the Dean McGee Eye Institute, Department of Ophthalmology at the University of Oklahoma Health Sciences Center, where I currently reside. My research is focused on corneal trauma, corneal dystrophies, and the development of noninvasive therapeutic agents. Throughout my career, I have given priority to interdisciplinary, translationally relevant approaches. I strongly believe that science is moving towards embracing these approaches and that they are key for future success.

I would like to apply some of those approaches, as we move forward with revamping ACP. A journal is only as good as its editorial team and the quality of its reviewers. Hence, building and maintaining a strong team of editors alongside a supporting team of editorial staff are the minimum requirements for a successful journal. My vision for ACP, as we move into this new chapter, is to attract contributing authors that will provide us global recognition and ensure that our journal is competitive, exciting, and useful for the scientific community. I, therefore, will do what I can in order to raise the journal's profile, maintain high standards in our publications, and increase our reach. Excitingly, our recent impact factor has been released (Figure 1) and shows consistent annual improvement. The previous leadership has set high standards that we can build on, and I am confident that we can improve further.  Figure 2 provides a summary of how many manuscripts were received, accepted, and rejected since 2016, highlighting our continuous and steady growth.

In terms of ACP's aim and scope, as a multidisciplinary, translational research journal, our focus should be on topics and areas that scientists, clinicians, and industry have prioritized. We have therefore updated those statements and will soon be available on our website.

The field of cellular pathology is broad and involves a whole spectrum of clinicians, scientists, and engineers. Our journal faces several challenges, which we will try and navigate through together, in the coming years. Perhaps the most significant is convincing enough young people to enter the field of cellular pathology and contribute to future discoveries and treatments. With translational medicine becoming popular and human studies a must, our journal has to attempt to lead the field to the new era. This approach will be one of the strategies, if not the only one, that will keep us competitive and allow us to grow as a unit.

The renewed ACP will focus on cross- and multidisciplinary publications that have the potential to revolutionize treatment of diseases. We will actively create opportunities for all those who are interested to submit and publish their work with us. The team and I are pushing for progress, we are pushing for excellence, and we will need all the help we can get from our Editorial Board members. In this era, communication is critical, and we will do what we can to keep the journal visible in medical and scientific communities. I understand that change is always difficult, but the challenges are too great and we must embrace the future.

In closing, I would like to extend my thanks to the team at Hindawi for this wonderful opportunity. I look forward to working with each team member in the future. On a more personal note, I would like to thank friends and colleagues who have always been very supportive of my decision to assume this role. I will continue to look to each of you for guidance and support as I begin this new task.

I am very excited about the future of ACP, and I look forward to working with our editorial team, the authors, the reviewers, and our readership as we continue to grow as a journal.

*Dimitrios Karamichos*

## Figures and Tables

**Figure 1:**
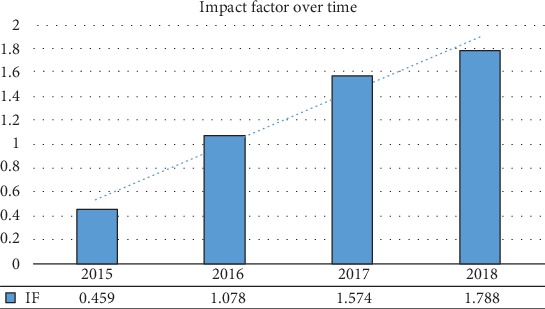
Impact factor (IF) of Analytical Cellular Pathology (ACP) journal, from 2015 to 2018. ACP, over the years, shows continued upward trajectory and should continue to improve.

**Figure 2:**
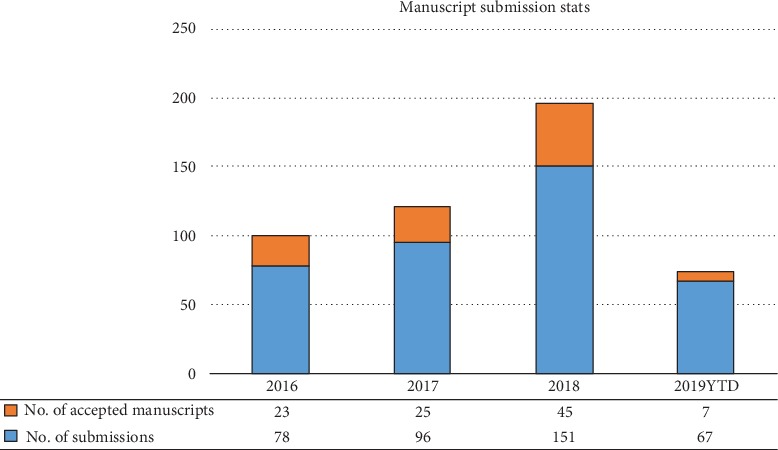
The number of manuscripts submitted to the Analytical Cellular Pathology (ACP) journal is shown from 2016 to 2018. 2019 data is correct as of June 2019.

